# Prevalence and Antimicrobial Susceptibility Profile of *Salmonella* and *Shigella* among Diarrheic Patients Attending Selected Health Facilities in Addis Ababa, Ethiopia

**DOI:** 10.1155/2023/6104416

**Published:** 2023-10-14

**Authors:** Tiruneh Ararsa, Deneke Wolde, Haile Alemayehu, Ketema Bizuwork, Tadesse Eguale

**Affiliations:** ^1^Tikur Anbesssa Specialized Hospital, College of Health Sciences, Addis Ababa University, Addis Ababa, Ethiopia; ^2^Aklilu Lemma Institute of Pathobiology, Addis Ababa University, Addis Ababa, Ethiopia; ^3^Department of Medical Laboratory Science, College of Medicine and Health Sciences, Wachemo University, Hossana, Ethiopia; ^4^The Ohio State University, Global One Health LLC, Addis Ababa, Ethiopia

## Abstract

Diarrhea is one of the important public health problems in developing countries. *Salmonella* and *Shigella* species are the major bacterial causal agents of diarrhea. The increasing burden of antimicrobial resistance is posing difficulty in the treatment of these pathogens. This study aimed to assess the occurrence of *Salmonella* and *Shigella* in the feces of diarrheic patients receiving health services in Addis Ababa, Ethiopia, and to determine their antimicrobial susceptibility profile. A cross-sectional study involving 13 health centers was conducted where 428 diarrheic patients were recruited. Standard microbiology techniques were used to isolate *Salmonella* and *Shigella* from stool samples. In addition, *Salmonella* isolates were confirmed by polymerase chain reaction (PCR). The Kirby–Bauer disc diffusion method was employed to assess susceptibility to 11 antimicrobials for each of the *Salmonella* and *Shigella* isolates. The prevalence of *Salmonella* and *Shigella* spp. among diarrheic patients was 8.4%; *n* = 36 and 5.6%; *n* = 24, respectively. Thirty (83.3%) of *Salmonella* isolates were susceptible to all antimicrobials tested, whereas 4 (10.8%) of isolates were resistant to 2 or more antimicrobials and 2 (5.6%) were multidrug resistant. Resistance to ampicillin was recorded in only one (2.7%) of *Salmonella* isolates; however, resistance to ampicillin was recorded in 12 (50%) of the *Shigella* isolates. Half of the *Shigella* isolates (*n* = 12) were resistant to 2 or more antimicrobials while 5 (20.8%) of them were resistant to 3 or more antimicrobials. The overall rate of resistance to antimicrobials was more common in *Shigella* compared to *Salmonella* isolates. In conclusion, *Salmonella* and *Shigella* were isolated from the feces of diarrheic patients, with a higher rate of antimicrobial resistance in *Shigella* isolates, which could make the treatment of shigellosis challenging. Therefore, increasing hygienic practices during food preparation to reduce the burden of *Salmonella* and *Shigella* infection and prudent use of antimicrobials are recommended to limit the spread of antimicrobial resistant strains.

## 1. Introduction

Diarrheal disease is among the principal causes of illness and death globally, especially in developing countries. It is one of the major causes of mortality particularly among children under the age of five years [1, 2]. The burden is high in sub-Saharan African countries. An estimated 1.8 million people die from diarrheal cases in developing countries per year due to a lack of improved sanitary facilities and limited access to clean drinking water [2–5]. Diarrheal disease is caused by a wide variety of bacterial, viral, and parasitic pathogens. Non-typhoidal *Salmonella*, *Shigella*, enterotoxigenic *Escherichia coli* (ETEC), *Campylobacter jejuni*, and *Vibrio cholerae* are the major bacterial etiologies of diarrhea in developing countries [6]. A single or multiple microbial agents could cause diarrheal diseases [7]. *Salmonella* and *Shigella* spp. are major causes of acute bacterial gastroenteritis and diarrheal diseases in developing countries [3, 8, 9]. Non-typhoidal *Salmonella* causes 93.8 million diarrheal cases and more than 155,000 losses of human life each year [10]. On the other hand, an estimated 165 million diarrhea episodes occur due to *Shigella* alone every year [11].

Antibiotics have shown benefits in shortening the duration and reducing the severity of diarrheal illness in patients infected with *Shigella* and in a subset of patients with non-typhoidal *Salmonella*. However, antimicrobial resistance in *Salmonella* and *Shigella* species is rapidly increasing globally, particularly in developing countries, where the use of antimicrobials in humans and animals is largely unrestricted. Treatment is mainly based on clinical findings because of a lack of laboratory facilities [12]. *Salmonella* and *Shigella* spp. are on the WHO list of antibiotic-resistance “priority pathogens” at high and medium threat levels, respectively [13].

Despite several efforts, diarrheal disease in Ethiopia remains elusive with high levels of morbidity and mortality. Non-typhoidal *Salmonella* serovars and *Shigella* species are among the commonly recovered diarrheagenic pathogens from diarrheal cases in the country [14, 15]. On the other hand, the rise of resistance to multiple antimicrobials among *Salmonella* and *Shigella* is becoming a serious global concern. Although *Salmonella* and *Shigella* are known to contribute a significant burden of morbidity and mortality globally, recent data on their occurrence and antimicrobial susceptibility profile in Addis Ababa are lacking. This study, therefore, was designed to assess the occurrence of *Salmonella* and *Shigella* among diarrheic patients and their susceptibility profile to selected antimicrobials.

## 2. Materials and Methods

### 2.1. Study Area and Design

The study was conducted in Addis Ababa from August 1, 2020, to August 31, 2021. Addis Ababa is the Capital of Ethiopia and the diplomatic center of Africa. It is located in the geographic epicenter of the country. Its average altitude is 2,400 meters above sea level, with the highest elevation at Entoto Hill to the north reaching 3,200 meters. This makes Addis Ababa one of the high-altitude capital cities of the world. The city is a home to 25% of the country's urban population. According to the Central Statistical Agency of Ethiopia, Addis Ababa had an estimated population of over 3.7 million in 2020 [16]. The city is divided into 11 subcities. A health facility-based cross-sectional study was conducted among diarrheic patients attending public health facilities in Addis Ababa.

### 2.2. Sample Size, Study Population, and Sampling

The sample size was determined using a single population proportion formula considering the following assumptions: 95% confidence level, 5% margin of error, 18.1% prevalence from previous study [14], and design effect of 1.5 and 10% nonresponse. Finally, the minimum calculated sample size was 394. However, in order to increase precision, a total of 428 diarrheic patients were involved in the study. A multistage sampling method was used to select study participants. First, four subcities: Kolfe Keranyo, Lideta, Addis Ketema, and Arad, were randomly selected out of eleven subcities in Addis Ababa. Then, thirteen Health Centers were involved from these four subcities. From Kolfe Keranyo subcity: Kolfe, Lomimeda, and Woreda 11 Health centers, from Lideta subcity: Abinet, Teklehymanot, and Lideta Health Centers, from Addis Ketema subcity: Kuas Meda, Abebe Bikila, Millennium, and Addis ketema Health Centers, and from Arada subcity: Arada, Janmeda, and Kebena Health Centers were randomly selected. The sample size was proportionally allocated to each health facility according to patient flow. Consecutive patients with symptoms of acute diarrhea were then recruited from each Health Center until the required number was fulfilled. One hundred seven diarrheic patients were targeted from each subcity. Criteria for inclusion of patients into the study were having symptoms of acute diarrhea whereas patients with persistent diarrhea and those who received antimicrobials within the last four weeks were excluded from the study. Diarrhea was defined as having loose or watery stools at least three times per day, or more frequently than usual for an individual [17].

### 2.3. Data and Sample Collection

A pretested structured questionnaire was used to collect sociodemographic (age, sex, and level of education), behavioral (consumption of raw milk and meat, toilet per a family, and hand washing behavior after using toilet), and clinical data from each study participants. All patients were provided with clean plastic stool collection cup with a tight stopper and oriented on how to collect stool specimen. Collected stool specimens were then transported in icebox containing ice pack to the Microbiology Laboratory of Aklilu Lemma Institute of Pathobiology, Addis Ababa University, within four hours of collection, and specimen was processed on the same day.

### 2.4. Culture and Identification of *Salmonella* and *Shigella*

One gram of stool specimen was mixed with 9 ml of buffered peptone water (BPW) (Oxoid, Basingstoke, UK) and incubated at 37°C for 24 hours. Then, a loopful of enriched suspension was plated on *Salmonella Shigella* agar (SSA) plate (Oxoid, Basingstoke, UK) and incubated for 24 hours at 37°C to isolate *Shigella*. For *Salmonella spp.* isolation, 100 *µ*l of pre-enriched suspensions was added into 9.9 ml of Rappaport–Vassiliadis enrichment broth (RVB) (Oxoid, Basingstoke, UK) and incubated at 42°C for 24 hours. At the same time, 1 ml of suspension was also transferred to 10 ml of tetrathionate broth (TTB) (Oxoid, Basingstoke, UK) and incubated for 24 h at 37°C. It was then streaked from both RVB and TTB to xylose lysine deoxycholate agar selective media (XLD) (Oxoid, Basingstoke, UK), and the plates were incubated at 37°C for 24 hours [18]. The growth of *Salmonella* and *Shigella* spp. was detected by their characteristic appearance on SS agar (*Salmonella*: black-centered colonies and *Shigella*: smooth and opaque or colorless) and XLD (*Salmonella* red with a black center and *Shigella*: red colonies). Suspected colonies were streaked onto tryptic soya agar slant (TSA) (Oxoid, Basingstoke, UK). Isolates were then further identified using different biochemical tests such as urea, triple sugar iron agar, citrate, lysine iron agar, and sulphide indole motility test. Isolates with typical biochemical test results of no urease production, ferment glucose, reduce sulfur, decarboxylate lysine, and positive for motility test were considered presumptive *Salmonella*. On the other hand, urease-negative, oxidase-negative, lysine decarboxylation-negative, and nonmotile isolates on motility-indol-ornithine agar with variable indol and ornithine activity were considered as *Shigella* species [19]. Presumptive *Salmonella* colonies with typical biochemical properties of *Salmonella* were further confirmed using genus-specific polymerase chain reaction [20].

Briefly, one to two pure colonies were picked and suspended in 100 *µ*l of nuclease-free water (ultra-pure DNase/RNase-free Distilled Water, Thermo Fisher, USA) in PCR tubes. It was then boiled for 5 min at 95°C in thermocycler, and an aliquot (1 *µ*l) of the supernatant was used as the template for PCR amplification. *Salmonella* Typhimurium (ATCC 14028) was used as a positive control. PCR reaction mix (20 *µ*l) consisting of 18 *µ*l nuclease-free water, 1 *µ*l of the template DNA, and 0.5 *µ*l of each of primers (reverse and forward) was prepared and added into PCR premix (AccuPower^@^Hot Start PCR PreMix, Korea) tubes containing 1U Taq DNA polymerase, 250 *µ*M dNTPs, and 1x reaction buffer with 1.5 mM MgCl_2._ The PCR amplification was performed using a thermal cycler with an initial denaturation (4 min at 95°C), 30 cycles of (denaturation at 95°C for 30 sec, annealing at 60°C for 30 sec, and extention at 72°C 45 sec), and final extension for 5 min at 72°C in a thermocycler. The amplified products were visualized by agarose gel electrophoresis, using 2% agarose gel stained with ethidium bromide. Positive results were confirmed by the presence of a 496-bp band seen on the gel with an ultraviolet transilluminator.

### 2.5. Antimicrobial Susceptibility Testing

Susceptibility testing to common antimicrobials was performed for 36 PCR confirmed *Salmonella* spp. and 24 isolates of *Shigella* confirmed by biochemical tests. Antimicrobial susceptibility was tested for 11 antimicrobials using Kirby–Bauer disc diffusion method according to Clinical and Laboratory Standards Institute Guidelines [21]. Morphologically identical colonies of overnight grown culture were suspended in 2 ml of normal saline, and turbidity was adjusted to 0.5 McFarland standard. It was then inoculated on Muller Hinton agar plate using a sterile cotton swab, and antimicrobial discs were then placed on plates keeping sufficient distance from each other on the medium and incubated at 37°C for 24 hours. The zone of inhibition was measured to the nearest millimeter using a caliper. Antimicrobial discs used were amoxicillin + clavulanic acid (20/10 *μ*g), amikacin (30 *μ*g), ampicillin (10 *μ*g), ceftriaxone (30 *μ*g), chloramphenicol (30 *μ*g), ciprofloxacin (5 *μ*g), gentamicin (10 *μ*g), nalidixic acid (30 *μ*g), tetracycline (30 *μ*g), sulfamethoxazole + trimethoprim (23.75 *μ*g/1.25 *μ*g), and streptomycin (10 *μ*g). Azithromycin (15 *μ*g) was tested only for *Shigella* isolates, and ceftriaxone was also tested only for *Salmonella* isolates. *E. coli* (ATCC 25922) was used as a quality control reference strain. The results were interpreted according to CLSI guidelines and recorded as sensitive (S), resistant (R), or intermediate (I) [21]. MDR is defined as nonsusceptibility to at least one agent in three or more antimicrobial categories and up to (and including) the total number of all antimicrobial categories minus two [22].

### 2.6. Data Analysis

Variables were summarized using descriptive statistics such as frequency, percentage, mean, and median considering the nature and distribution of the variables. The presence of a significant association between different variables and *Salmonella* and *Shigella* positivity was assessed using the Chi-square test or Fisher's exact test, as appropriate. Data analysis was carried out using SPSS version 23.

## 3. Results

### 3.1. Sociodemographic Characteristics and Prevalence of *Salmonella* and *Shigella*

**Figure 1:**
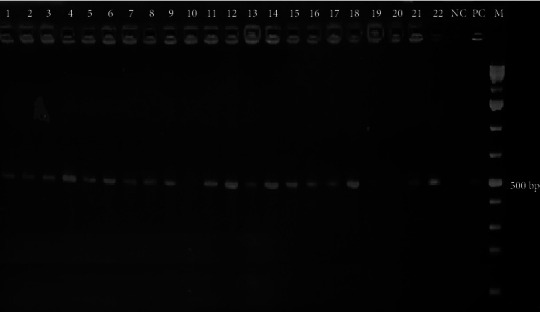
Representative gel image of suspected isolates confirmed by amplification of *Salmonella*genus-specific region of histidine transport operon. Lanes 1–9, 11–18, and 21-22 are positive isolates showing 496-bp amplified region; lanes 10, 19, and 20 were negative; lane 23 negative control; lane 24 positive control; M-1 kb plus molecular weight marker.

Two hundred thirty-six (55.1%) of the study participants were female. Twenty-five years of age or older accounted for 51.6% of the total participants. Over a quarter (26.9%) of the study participants had education to the college level and above. Raw milk consumption and the habit of consuming raw meat were reported by 86 (20.1%) and 49 (11.5%) of the participants, respectively. Representative image of genus-specific PCR gel image showing 496 base pair amplification of *Salmonella* isolates is shown in Figure 1. In this study, the overall prevalence of *Salmonella* and *Shigella* was 8.4% (*n* = 36) (95% CI 5.8, 11.2%) and 5.6% (*n* = 24) (95% CI 3.5, 7.9%), respectively. Out of the 24 patients positive for *Shigella*, 12 (50%) of them were older than 25 years of age, and none of the diarrheic children under one year of age were positive for *Shigella* spp. Age and sex of the patient, educational status, use of the shared toilet, and consumption of raw milk and meat were not significantly associated with the prevalence of *Shigella* and *Salmonella* among diarrheic patients in the current study. However, those patients who did not wash their hands after using the toilet were more likely to be tested positive for *Salmonella* from their stool sample (*p* = 0.022). There was no statistically significant difference in the prevalence of *Shigella* in patients consuming raw milk and meat and those not consuming whereas a relatively high occurrence of *Salmonella* was recorded in patients who consumed raw meat (12.2%) compared to those not consuming raw meat (7.9%). However, this difference was not statistically significant (Table 1).

### 3.2. Antimicrobial Susceptibility Profile of *Salmonella* and *Shigella* Isolates

Both *Salmonella* and *Shigella* species showed different resistance patterns to the antimicrobials tested. Overall, high rates of resistance to antimicrobials were observed in *Shigella* isolates compared to *Salmonella* isolates. *Shigella* isolates showed 50% resistance to ampicillin whereas only 2.7% of *Salmonella* isolates were resistant to the same antimicrobial agent. In addition, resistance to tetracycline was detected in 33.3% of *Shigella* and in 5.6% of *Salmonella* isolates. Resistance to amoxicillin + clavulanic acid was detected in 20.8% of *Shigella* isolates; however, none of the *Salmonella* isolates were resistant to this antimicrobial agent. *Salmonella* was found to have a low resistance rate (2.8%) to amikacin and gentamicin, while none of the *Shigella* isolates were completely resistant to these antimicrobials. All *Salmonella* species were sensitive to chloramphenicol, while 4.2% of *Shigella* isolates were resistant to this antimicrobial agent (Table 2).

### 3.3. Resistance Pattern of *Salmonella* and *Shigella* Isolates

Most (83.3%) of *Salmonella* isolates in this study were susceptible to most antimicrobial agents tested. Resistance to one or more antimicrobials was recorded in 83.3% of *Shigella* isolates and in 16.7% of *Salmonella* species. Only 2 (5.4%) of *Salmonella* isolates were resistant to 2 antimicrobials, and 2 (5.4%) isolates were resistant to 3 antimicrobials. On the other hand, multiple antimicrobial susceptibility patterns were recorded for *Shigella* isolates ranging from 4 isolates (16%) susceptible to all tested antimicrobials to a single isolate (4.2%) resistant to as high as 6 antimicrobials. Twelve (50%) of the *Shigella* isolates were resistant to 2 or more antimicrobials whereas 5 (20.8%) were resistant to ≥3 antimicrobials (MDR). All MDR *Shigella* species were resistant to tetracycline, and 80% of them were resistant to ampicillin and streptomycin. Six resistance patterns composed of five antimicrobial classes were detected in *Salmonella* species, while thirteen resistance patterns composed of eight antimicrobial classes were detected in *Shigella* (Table 3).

## 4. Discussion

This study determined the prevalence of *Salmonella* and *Shigella* and their susceptibility patterns to antimicrobials in patients attending public health facilities in Addis Ababa, Ethiopia. The prevalence of *Salmonella* and *Shigella* was 8.4% (95% CI 5.8, 11.2%) and 5.6% (95% CI 3.5, 7.9%), respectively. The prevalence of *Salmonella* recorded in the current study is in line with previous reports from various regions of Ethiopia: Addis Ababa, (7.2%) [18], Nekemte (7.1%) [17], and Kenya (10.4%) [23]. However, it is lower than reports from Adama (21.4%) [14] and Northern India (22.7%) [24]. The finding of this study is higher than reports from Bishoftu (2.3%) [25] and Ambo (1.3%) [15]. Furthermore, it is also higher than the report from young diarrheic children in Kenya (3.5%) [26], and this variation could be attributed to a difference in geographic location, year of the study, or difference in the distribution of predisposing factors for bacterial contamination such as consumption of raw meat in study population from different backgrounds.

The prevalence of *Shigella* in our study was comparable to a previous study conducted in Goba (4.3%) [27]. It is slightly higher than other studies conducted in Ambo (2.5%) [15], Jimma (2.3%) [16], and Nekemte (2.1%) [17, 28], and two studies conducted in Kenya (2%, 2.8%) [23, 26]. However, the prevalence is lower than the previous report from rural Coastal India (11.2%) [29]. The reason for this might be due to difference in sociodemographic characteristics of study participants. The current study involved all age groups, whereas some of the previous studies involved only under five children [17, 26] and children aged between 0 and 15 years [16]. In the Indian study, both inpatients and outpatients were included [29], whereas participants of this study were all outpatients with little history of hospitalization. The other possible explanation could be the difference in geographic location.

The rise and spread of antimicrobial resistance has become challenging to the treatment and control of infectious diseases [30]. This study detected a high resistance rate, particularly among *Shigella* isolates to selected antimicrobials such as ampicillin (50%), tetracycline (33.3%), amoxicillin + clavulanic acid (20.8%), streptomycin (20.8%), and sulfamethoxazole + trimethoprim (20.8%). A much higher resistance rate of *Shigella* isolates was reported from previous studies to ampicillin [14, 23] and tetracycline [14, 27]. The rate of antimicrobial resistance in *Salmonella* isolated in this study is low compared to the reports from previous study in the same area, particularly for antimicrobials in the classes of beta-lactams (ampicillin), tetracycline, and aminoglycosides [18]. The possible reason why resistance to *Shigella* is relatively high compared to *Salmonella* could be due to the excessive use of antimicrobials in the human population in the study area, and as *Shigella* is a host-specific pathogen circulating only among the human population through contaminated food and water [31], spread of resistant strains among human population is high. On the other hand, non-typhoidal *Salmonella* species are zoonotic, and they are transmitted via contaminated animal source food and produces through direct or indirect contact with food animals. The low rate of resistance in *Salmonella* could be due to less burden of antimicrobial resistance in animals due to less use of antimicrobials or due to less exposure to antimicrobial resistant *Salmonella*-contaminated food products among patients involved in this study. The indiscriminate use of antimicrobials results in the emergence of MDR *Salmonella* and *Shigella* spp. which makes their infection a global threat. The prevalence of MDR among *Salmonella* isolates in the current study 5.6% is lower than the findings of previous studies conducted in Nekemte (10%) [28] and Goba (31%) [27]. With regard to *Shigella* spp., the prevalence of MDR was 20.8% which is significantly lower than the rate of resistance previously reported in Goba (100%) [27], Cambodia (98%) [32], and China (91.1%) [33]. The discrepancy could be related to inappropriate or excessive use of antibiotics, which are major factors in antimicrobial resistance. Studies conducted in different parts of Ethiopia showed a high rate of multidrug resistance among *Salmonella* isolates. In these study areas, self-medication, dropping prescribed antimicrobials before their full course of therapy, and sharing medication with other people are common practices in the communities [34, 35]. The presence of a significant knowledge and practice gap on antimicrobial resistance among the general public, patients, and livestock producers is also reported recently [36].

## 5. Conclusion

High rate of *Salmonella* and *Shigella* spp. positivity was recorded from the stool of diarrheic patients, with a higher rate of antimicrobial resistance to commonly prescribed antibiotics among *Shigella* isolates. Clinicians must take this information into account in the selection of the best antimicrobial agents for empirical treatment of patients presenting with acute infectious diarrhea. There is also a need for infection prevention strategies and continuous antimicrobial susceptibility testing programs to tackle the problem.

## Figures and Tables

**Table 1:** Sociodemographic characteristics and prevalence of *Salmonella* and *Shigella* among diarrheic patients, *N* = 428.

Variables	No. tested	No. positive for *Shigella* spp. (%)	*X* ^2^	*p* value	No. positive for *Salmonella* spp. (%)	*X* ^2^	*p* value
Age in years							
<1	6	0 (0.00)	0.48	0.92	1 (16.7)	6.39	0.09
1–9	77	5 (6.5)			8 (10.4)		
10–24	124	7 (5.6)			4 (3.2)		
≥25	221	12 (5.4)			23 (10.4)		
Sex							
Male	192	10 (5.2)	0.01	0.46	17 (8.9)	0.02	0.45
Female	236	14 (5.9)			19 (8.1)		
Educational status							
Children below school age	70	6 (8.6)	2.9	0.430	7 (1.0)	0.57	0.90
Do not read and write	44	1 (2.3)			4 (9.1)		
1–12 Grade	199	9 (4.5)			17 (8.5)		
College and above	115	8 (7.0)			8 (7)		
Use shared toilet							
Yes	85	4 (4.7)	0.02	0.46	6 (7.1)	0.08	0.39
No	343	20 (5.8)			30 (8.7)		
Hand-washing behavior after using a toilet							
Yes	390	21 (5.4)	0.07	0.36	29 (7.4)	4.1	0.02
No	38	3 (7.9)			7 (18.4)		
Consumption of raw milk							
Yes	86	5 (5.8)	0.08	0.55	3 (3.8)	2.6	0.04
No	342	19 (5.6)			33 (9.5)		
Consumption of raw meat							
Yes	49	3 (6.1)	0.03	0.53	6 (12.2)	0.57	0.23
No	379	21 (5.5)			30 (7.9)		
Total	428	24 (5.6)			36 (8.4)		

**Table 2:** Antimicrobial susceptibility profile of *Salmonella* and *Shigella* isolates.

Antibiotics pattern	*Salmonella* (*n* = 36)			*Shigella* (*n* = 24)		
	*S*	*I*	*R*	*S*	*I*	*R*
AM	32 (88.8)	3 (8.3)	1 (2.7)	11 (45.8)	1 (4.2)	12 (50)
AMC	35 (97.2)	1 (2.8)	0 (0)	16 (66.6)	3 (12.5)	5 (20.8)
AN	32 (88.88)	3 (8.3)	1 (2.8)	21 (87.5)	3 (12.5)	0 (0)
C	36 (100)	0 (0)	0 (0)	23 (95.8)	0 (0)	1 (4.2)
CIP	25 (69.44)	11 (30.6)	0 (0)	13 (54.16)	10 (41.67)	1 (4.2)
CRO	29 (80.6)	6 (16.7)	1 (2.8)	NT	NT	NT
GM	14 (38.9)	21 (58.3)	1 (2.8)	6 (25)	18 (75)	0 (0)
NA	34 (94.4)	1 (2.8)	1 (2.8)	19 (79.2)	0 (0)	5 (20.8)
TE	34 (94.44)	0 (0)	2 (5.6)	15 (62.5)	1 (4.2)	8 (33.3)
S	8 (22.2)	26 (72.2)	2 (5.6)	3 (12.5)	16 (66.7)	5 (20.8)
SXT	35 (97.2)	0 (0)	1 (2.8)	17 (70.88)	2 (8.3)	5 (20.8)
AZM	NT	NT	NT	22 (91.66)	0 (0)	2 (8.3)

*Note*. AM: ampicillin, AMC: amoxicillin + clavulanic, AN: amikacin, C: chloramphenicol, CIP: ciprofloxacin, CRO: ceftriaxone, GM: gentamicin, NA: nalidixic acid, TE: tetracycline, S: streptomycin, SXT: sulfamethoxazole + trimethoprim, AZM: azithromycin, and NT: not tested.

**Table 3:** Antimicrobial resistance pattern of *Salmonella* and *Shigella* isolates.

Resistant pattern	No. of isolates with this resistance pattern (%)	Resistant to how many antimicrobials
*Salmonella* (*N* = 36)		
—	30 (83.3)	0
Am	1 (2.8)	1
An	1 (2.8)	1
Am, Te	1 (2.8)	2
S, Na	1 (2.8)	2
Am, S, Sxt	1 (2.8)	3
Am, Cro, Te	1 (2.8)	3
*Shigella* (*N* = 24)		
—	4 (16)	0
Am	3 (12.5)	1
Azm	2 (8.3)	1
NA	3 (12.5)	1
Te	1 (4.2)	1
Am, Amc	3 (12.5)	2
Am, SXT	1 (4.2)	2
Am, Te	1 (4.2)	2
C, Te	1 (4.2)	2
Am, S, Te	1 (4.2)	3
S, Te, STX	1 (4.2)	3
Am, Amc, S, Te, SXT	1 (4.2)	5
Am, S, Te, Na, SXT	1 (4.2)	5
Am, Amc, S, Te, Na, SXT	1 (4.2)	6

Am: ampicillin; Amc: amoxicillin + clavulanic acid; An: amikacin; C: chloramphenicol; Cro: ceftriaxone; Na: nalidixic acid; Te: tetracycline; S: streptomycin; SXT: sulfamethoxazole + trimethoprim; Azm: azithromycin.

## Data Availability

The data supporting the current study are available from the corresponding author upon request.

## Abbreviations

AMR: Antimicrobial resistance
BPW: Buffered peptone water
NTS: Nontyphoidal *Salmonellae*
RVB: Rappaport–Vassiliadis enrichment broth
SSA: *Salmonella shigella* agar
TTB: Tetrathionate broth
TSA: Tryptone soya agar
XLD: Xylose lysine deoxycholate agar.
